# Chain of care for patients who have attempted suicide: a follow-up study from Bærum, Norway

**DOI:** 10.1186/1471-2458-11-81

**Published:** 2011-02-04

**Authors:** Håkon A Johannessen, Gudrun Dieserud, Diego De Leo, Bjørgulf Claussen, Per-Henrik Zahl

**Affiliations:** 1 Department of Suicide Research and Prevention, Division of Mental Health, Norwegian Institute of Public Health, Oslo, Norway; 2 Australian Institute for Suicide Research and Prevention, Mt Gravatt Campus, GriffithUniversity, Brisbane, Australia; 3 Institute of General Practice and Community Health, Section for Social Medicine, University of Oslo, Oslo, Norway

## Abstract

**Background:**

Individuals who have attempted suicide are at increased risk of subsequent suicidal behavior. Since 1983, a community-based suicide prevention team has been operating in the municipality of Bærum, Norway. This study aimed to test the effectiveness of the team's interventions in preventing repeated suicide attempts and suicide deaths, as part of a chain of care model for all general hospital treated suicide attempters.

**Methods:**

Data has been collected consecutively since 1984 and a follow-up was conducted on all individuals admitted to the general hospital after a suicide attempt. The risk of repeated suicide attempt and suicide were comparatively examined in subjects who received assistance from the suicide prevention team in addition to treatment as usual versus those who received treatment as usual only. Logistic regression and Cox regression were used to analyze the data.

**Results:**

Between January 1984 and December 2007, 1,616 subjects were registered as having attempted suicide; 197 of them (12%) made another attempt within 12 months. Compared to subjects who did not receive assistance from the suicide prevention team, individuals involved in the prevention program did not have a significantly different risk of repeated attempt within 6 months (adjusted *OR *= 1.08; 95% CI = 0.66-1.74), 12 months (adjusted *OR *= 0.86; 95% CI = 0.57-1.30), or 5 years (adjusted *RR *= 0.90; 95% CI = 0.67-1.22) after their first recorded attempt. There was also no difference in risk of suicide (adjusted *RR *= 0.85; 95% CI = 0.46-1.57). Previous suicide attempts, marital status, and employment status were significantly associated with a repeated suicide attempt within 6 and 12 months (p < 0.05). Alcohol misuse, employment status, and previous suicide attempts were significantly associated with a repeated attempt within 5 years (p < 0.05) while marital status became non-significant (p > 0.05). With each year of age, the risk of suicide increased by 3% (p < 0.05).

**Conclusions:**

The present study did not find any differences in the risk of fatal and non-fatal suicidal behavior between subjects who received treatment as usual combined with community assistance versus subjects who received only treatment as usual. However, assistance from the community team was mainly offered to attempters who were not receiving sufficient support from treatment as usual and was accepted by 50-60% of those deemed eligible. Thus, obtaining similar outcomes for individuals, all of whom were clinically judged to have different needs, could in itself be considered a desirable result.

## Background

Attempted suicide is a serious public health problem. The National Co-morbidity Study reported a lifetime prevalence of attempted suicide of 4.6% [1], a figure substantially similar to the one derived from a large Australian community survey (4.2%) [2]. Furthermore, the risk of subsequent suicidal behavior is substantial: about 15% of those who attempt suicide had another attempt within one year [3]. The suicide risk for males and females who have previously attempted suicide is reportedly 55 and 77 times that of the general male and female population, respectively [4]. Consequently, effective after-care strategies aimed at individuals who have attempted suicide are important [5].

Although various psychosocial and pharmacological treatments in preventing subsequent suicidal behavior have been developed, there is a lack of sufficient scientific evidence of efficacy for the various strategies used [6-9]. Due to the low rate of suicide, it is difficult to test the effectiveness of intervention strategies that aim to prevent suicide among suicide attempters [9-11]. For example, given that the risk of committing suicide in this group is 2.8%, Gunnell and Frankel [6] estimated that a total sample size of almost 45,000 subjects would be needed to demonstrate a 15% reduction of suicide risk. Therefore, a repeated suicide attempt has been used as a reasonable proxy because it occurs more frequently and it is strongly related to successful suicide [9-12]. Further, a repeated suicide attempt is obviously an outcome that should be prevented - it indicates persistent distress for the individual and results in considerable health care costs [9]. Small sample size has been a frequent issue in studies evaluating repeated suicide attempts as an outcome measure. Consequently, a very limited number of investigations have managed to detect clinically meaningful and statistically significant effects of suicide interventions [9].

Recently, Hvid and Wang [13] published a study on the effectiveness of an outreach intervention aimed at individuals who had attempted suicide. They found that individuals in the intervention group who were offered follow-up care by a rapid response outreach program had a lower rate of repeated suicide attempts than controls (*RR *= 0.43; 95% CI = 0.23-0.80). Other studies have indicated potential beneficial effects of interventions that include active outreach and contact maintenance on a regular basis after attempted suicide [14-17]. In Norway, a suicide intervention model, similar to the one studied by Hvid and Wang, has been in effect since 1983 in a municipality outside the capital. This intervention is community-based and flexibly serves the individual by creating a multi-targeted, coordinated, and long lasting chain of care. The objective of the present study was to investigate the risk of repeated suicide attempt and successful suicide in individuals assisted by a suicide prevention team organization versus individuals who received treatment as usual without assistance from the suicide prevention team.

## Methods

### The Bærum Model

A rapid-response intervention is created through collaboration between the general hospital of Asker and Bærum, the municipal suicide prevention team, and community health and social services located in the municipality of Bærum. On presentation at the hospital or emergency unit, patients receive acute life-saving treatment and medical monitoring. Consequently, a hospital-based suicide prevention team, including social workers and a liaison psychiatrist, is notified. This team helps the patients through crisis intervention and evaluates the patients' psychosocial functioning and risk for suicide. Appropriate measures are then taken with the patients' cooperation. These measures can include referrals to psychiatric inpatient and various outpatient services, including mental health treatment, substance abuse treatment, family counseling, and various social services.

All patients that are not immediately admitted to psychiatric inpatient treatment are evaluated for referrals to the community suicide prevention team. A minority of the patients admitted to inpatient mental health treatment receives community suicide prevention team services (n = 28 in the present study). The community team and the hospital team collaborate to ensure a joint evaluation of the situation, to make appropriate referrals, and to ensure that all follow-up steps are in effect as soon as possible. Main patient groups eligible for referrals to the suicide prevention team are as follows:

• Patients in need of outpatient health and social services that are not established by the hospital team

• Patients in ongoing outpatient treatment who are in need of extra support

• Patients and family or other who are in relational conflicts and in need of extra support

• Patients who have previously dropped out of mental health treatment and need to be motivated to reappoint

The community suicide prevention team includes public health nurses and a psychologist. The nurses organize the work in relation to patients, in consultation with the psychologist. Particular emphasis is placed on the suicidal person's need for a supportive helper. On average, 50 - 60% of the attempters deemed eligible for the community team are referred to this follow-up every year.

If patients agree to be assisted by this team, a public health nurse contacts them shortly after discharge. A telephone call is made, preferably the day of discharge or the day after. An agreement is made on when and where to meet; in most cases the agreement involves nurse making a home visit within a few days. These nurses act as "ombudsmen" [18]: the main aim is to ensure that the patients are given sufficient follow-up care by specialist mental health services or community health/social services within an acceptable period of time following hospital discharge. Further, the nurse motivates the patient to accept treatment and better adhere to treatment appointments. If patients drop out of treatment, the nurse tries to recuperate them back into treatment or establishes a more suitable therapeutic plan in collaboration with the patient and the health services.

In addition, the nurse fulfills the role of looking after the patient between hospital discharge and established aftercare. The nurse gives the patient "psychological first aid", problem-solving counseling, and activates the patient's social network. Although the main aim is reached when a treatment program is established, the patient is followed-up by the nurse for approximately one year to secure continuity, treatment compliance, and social support. Most of the contact will be phone calls, ranging from several times a week in the beginning, to a monthly call at the end of the follow-up period.

The intervention offered by this team is not regarded as stand-alone treatment, but rather as a service offered in addition to established health and social services; it is not a substitute for any other health interventions. If the standard follow-up treatment is deemed to be sufficient, the community team is not activated. Patients are in any case free to reject assistance from the community team.

### Data

The data set is comprised of unselected individuals, who were residents of the municipality of Bærum and admitted to the general hospital after a suicide attempt between 1^st ^January 1984 and 31^st ^December 2008. The municipality of Bærum is a suburb located on the outskirts of Oslo, the capital of Norway [19]. Compared to the national average, Bærum is characterized by a higher population density, a higher income and education level for both men and women, and a lower unemployment rate. In 1984, the population of Bærum, aged above 15 years, was 71,237 (34,306 males and 36,931 females); in 2008, it was 80,368 (38,463 males and 41,905 females).

Data were collected in two stages. Firstly, data on individuals who had made a suicide attempt were consecutively collected from 1984 [20] using records from the local hospital in Bærum and the Municipal Health Services office. A quality control auditing was performed by rechecking all entered data. The definitional criteria for 'attempted suicide' were similar to those of 'parasuicide' used in the WHO/EURO Multicenter Study on Suicidal Behavior: "An act with nonfatal outcome, in which an individual deliberately initiates a non-habitual behavior that, without intervention from others, will cause self-harm, or deliberately ingests a substance in excess of the prescribed or generally recognized therapeutic dosage, and which is aimed at realizing changes which the subject desired via the actual or expected physical consequences"[21]. At the general hospital, an alert system, effective since 1984, ensures that all patients treated in the hospital unit and A & E unit that were admitted after intoxications regardless of intention, as well as all suicide related injures, are reported to the hospital suicide prevention team. This team is involved in classification and intervention. Inclusion in the suicide attempt database is made according to the above-mentioned definition, and calculations of repetitions are made from this database. Further, the liaison at the hospital and the psychologist in the community health services continuously recheck each case to ensure correct inclusion of cases. The authors of the present paper are blind to the intervention history of the patients.

Secondly, data on mortality of all causes were collected by linking the personal identifier of individuals who had made a suicide attempt to the computerized Causes-of-Death Registry at Statistics Norway. This registry has an almost complete registration of causes of death. Prior to 1986, causes of deaths were classified according to the International Classification of Diseases Eight Revision (ICD-8), from 1986-1995 according to the ICD-10, and from 1996 onwards according to the ICD-10. The last date of mortality information in the present data set was 31 December 2003.

The outcome variable 'repeated suicide attempt' was defined as a new record of suicide attempt within 6 months, 12 months, and 5 years after the index attempt. The index attempt refers to the first recorded suicide attempt. In order to be recorded, the reattempt had to result in emergency unit or hospital admittance in Bærum general hospital. Variables entered into the analyses at the time of the index attempt were: suicide prevention team assistance, age, sex, mental health aftercare referrals, alcohol misuse, marital status, employment status, and previous suicide attempts. The classification of these variables was based on information from medical records and information given by patients when interviewed at the hospital or by personnel in the community health services. Data on persons attempting suicide were consecutively collected at the hospital, and separate records were kept for each individual. The second author (GD) was responsible for data quality and consistency throughout the study period, and has completed the forms, one per person, together with the liaison psychiatrist at the hospital. Calculations on repetition were made based on these records.

The variable 'aftercare referrals' provides information on the type of standard follow-up treatment given to the individual; it also functions as a proxy measure of the kind of problems the attempter is presenting. In the regression analysis, psychiatric out-patient services, addiction services, and family services were grouped together and labeled as 'Psychiatric outpatient services'; psychiatric hospital care was labeled as 'Psychiatric hospital care'; and social services, child care, home care, private practice psychotherapists, and other forms of care were grouped together and labeled as 'Other'.

All Norwegian citizens that are identified as ill have the right to adequate health care treatment, as stated by Norwegian health authorities. As a basic principle, health care treatment is voluntary. The patient group that did not receive any standard aftercare may have been evaluated as not in need of specific aftercare or may have refused the recommended treatment. The main motive for referrals to the suicide prevention team, which is not defined as health care treatment in terms of Norwegian law, is as follows: all patients who are admitted to the general hospital or emergency unit due to an attempted suicide are eligible to be offered assistance by a rapid outreach suicide prevention team, organized by the community health services, except patients that immediately after discharge are admitted to inpatient psychiatric care. Assistance from the suicide prevention team is voluntary; hence, patients are free to refuse it.

### Statistics

Logistic regression analyses were computed to estimate the risk for a repeated suicide attempt within 6 and 12 months following the first recorded suicide attempt. Cox regression analyses were computed to estimate the risk for a repeated suicide attempt within 5 years of follow-up and to estimate the risk for suicide. In the Cox regression analyses, the individuals were followed for different lengths of time. When the outcome was a repeated suicide attempt, the period of risk was estimated in months from the date of the first recorded suicide attempt to the occurrence of a new attempt, or the end of the five year follow-up period or end of the study (December 2008), whichever happened first. When the outcome was suicide, the period of risk was estimated in months from the date of the first recorded suicide attempt to death by all causes or to the end of study (December 2003), whichever happened first.

Permission to perform this study was obtained from the Norwegian Data Inspectorate, The Regional Committee for Medical Research Ethics, and the Norwegian Directorate of Health. Further, a Data Handling Treaty between the Hospital of Asker & Bærum and the Norwegian Institute of Public Health was obtained.

## Results

Between January 1984 and December 2007, 1,616 individuals were registered as having a suicide attempt. Of these, 197 (12.2%; 95% CI = 10.6-14.0) repeated the attempt within 12 months of follow-up. Among the 1,616 individuals, 1,311 were eligible for evaluation to be referred to the suicide prevention team. Information on whether these received assistance from the suicide prevention team was recorded for 1,304 individuals (missing = 7); 675 (52%) received the assistance (Table 1 and 2). Of these, 531 (79%) were also referred to other types of health or social services, while 140 (21%) were not referred to such services (missing = 4). Among the 629 (48%) individuals who did not receive assistance from the team, 435 (69%) were referred to other types of health or social services, while 172 (27%) were not referred to such services (missing = 22) (Table 2).

**Table 1:** Repeated suicide attempts within 12 months by gender and suicide prevention team

	Men		Women		Total	
Suicide prevention team	*N*	Cases (%)	*N*	Cases (%)	*N*	Cases (%)
Yes	182	26 (14)	493	54 (11)	675	80 (12)
No	231	20 (9)	398	50 (13)	629	70 (11)
Total	413	46 (11)	891	104 (12)	1304	150 (12)

**Table 2:** Characteristics of the index attempt by suicide prevention team

	No suicide prevention team assistance		Suicide prevention team assistance		Total
Variables	*N *= 629; 48%		*N *= 675; 52%		*N *= 1304
Aftercare referrals					
None	172	55%	140	45%	312
Psychiatric out-patient	111	44%	143	56%	254
Addiction services	63	39%	100	61%	163
Family services	35	24%	109	76%	144
Social services	6	18%	27	82%	33
Home care	61	78%	17	22%	78
Private Psychotherapist	47	42%	66	58%	113
Child care	34	64%	19	36%	53
Other	78	61%	50	39%	128
Missing	22	85%	4	15%	26
Sex					
Male	231	56%	182	44%	413
Female	398	45%	493	55%	891
Missing	0	0%	0	0%	0
Age					
15-19	99	44%	124	56%	223
20-29	150	46%	177	54%	327
30-39	120	51%	117	49%	237
40-49	103	45%	129	55%	231
50-59	61	48%	67	52%	128
60+	95	60.5%	62	39.5%	157
Missing	1	100%	0	0%	1
Previous attempts					
None	339	42%	462	58%	801
One	93	42%	128	58%	221
Several	40	38%	65	62%	105
Missing	157	89%	20	11%	177
Alcohol misuse					
No	513	49.5%	524	50.5%	1037
Yes	116	43%	151	57%	267
Missing	0	0%	0	0%	0
Marital status					
Married/cohabiting	195	42%	273	58%	468
Single	243	49%	250	51%	493
Widow(er)	34	65%	18	35%	52
Divorced/separated	84	39%	131	61%	215
Missing	73	96%	3	4%	76
Employment status					
Employed	145	32%	307	68%	452
Unemployed/disabled	91	42%	128	58%	219
Student/pupil	88	43%	117	57%	205
Others	112	55%	92	45%	204
Missing	193	86%	31	14%	224

Among the 675 individuals who received assistance from the suicide prevention team, 60 (9%) repeated an attempt within 6 months of their index episode, and 80 (12%) repeated within 12 months; 47 (7%) of those who did not receive assistance from the team repeated within 6 months and 70 (11%) repeated within 12 months (Table 1). As seen in Table 3, the odds ratio (OR) of a repeated suicide attempt within 6 months (adjusted OR = 1.14) and 12 months (adjusted OR = 0.93) were not significantly associated with the suicide prevention team variable or other aftercare options.

**Table 3:** Logistic regression analysis predicting repeated suicide attempt within 6 and 12 months after the index attempt

	Repeated suicide attempt within 6 months				Repeated suicide attempt within 12 months			
		
Variable	Crude analysis		Adjusted analysis		Crude analysis		Adjusted analysis	
	*OR*	95% CI	*OR*	95% CI	*OR*	95% CI	*OR*	95% CI
*Sex*								
Female	1		1		1		1	
Male	1.15	0.76-1.75	1.37	0.84-2.23	0.95	0.65-1.37	1.00	0.65-1.56
*Age*	1.01	1.00-1.02	1.00	0.98-1.02	1.01	1.00-1.02	1.00	0.98-1.02
*Suicide prevention team*								
No	1		1		1		1	
Yes	1.21	0.81-1.80	1.08	0.66-1.74	1.07	0.76-1.51	0.86	0.57-1.30
*Previous attempt*								
No	1		1		1		1	
One	1.41	0.84-2.36	1.41	0.80-2.48	1.37	0.88-2.15	1.30	0.79-2.11
Several	2.48*	1.38-4.44	2.60*	1.38-4.87	2.60*	1.56-4.33	2.52*	1.45-4.41
*Aftercare referrals*								
Others	1		1		1		1	
Out-patient	1.24*	1.02-1.52	1.25	0.78-2.01	1.59*	1.13-2.25	1.15	0.94-1.42
*Alcohol misuse*								
No	1		1		1		1	
Yes	1.66*	1.07-2.59	1.02	0.58-1.79	1.87*	1.28-2.73	1.17	0.72-1.90
*Marital status*								
Others	1		1		1		1	
Married/cohabitant	0.66	0.43-1.02	0.56*	0.34-0.94	0.74	0.51-1.07	0.62*	0.40-0.96
*Employment*								
Employed	1		1		1		1	
Unemployed/disabled	1.22	0.73-2.06	0.96	0.54-1.72	1.22	0.77-1.92	1.00	0.60-1.66
Student/pupil	0.49*	0.24-0.99	0.35*	0.15-0.81	0.45*	0.24-0.84	0.38*	0.18-0.79
Others	0.92	0.52-1.62	1.05	0.53-2.11	1.00	0.61-1.61	1.07	0.59-1.93

* p < 0.05

Previous suicide attempts, marital status, and employment status were significantly associated with a repeated suicide attempt within both 6 and 12 months (p < 0.05) (Table 3). Individuals who made several suicide attempts prior to their index attempt had a more than doubled odds ratio of subsequent suicide attempt within both 6 months and 12 months. Being married/cohabitant and being a student/pupil were associated with a significantly reduced odds ratio of a subsequent suicide attempt within both 6 and 12 months.

Data were also analyzed separately for men and women. Neither men nor women who received assistance from the suicide prevention team had a reduced adjusted odds ratio of a repeated suicide attempt (suicide attempt repeat within 12 months for women: adjusted OR = 0.69; p < 0.14).

The suicide prevention team's working routines were reshaped and improved in 1998. Therefore we studied if there were any differences following this change by analyzing the periods 1984-1997 and 1998-2007 separately. No significant differences were found. Finally, we analyzed the odds of a third suicide attempt being made within 12 months from the second attempt. Again, no differences were detectable in relation to the suicide prevention team variable (adjusted OR = 1.37; p > 0.05).

In Table 4, Cox regression analyses were computed in order to determine if the suicide prevention team influenced suicide attempt repetition within a 5-year follow-up period. Receiving assistance from the suicide prevention team was not significantly associated with a repeated suicide attempt (adjusted RR = 0.90; p > 0.05). In general, being a student/pupil significantly reduced the risk (adjusted RR = 0.47; p < 0.05); several previous suicide attempts (adjusted RR = 2.20; p < 0.05) and alcohol misuse (adjusted RR = 1.59; p < 0.05) increased the risk.

**Table 4:** Cox regression analysis predicting repeated suicide attempt within 5 years after the index attempt

Variable	Crude analysis		Adjusted analysis	
	*RR*	95% CI	*RR*	95% CI
*Sex*				
Female	1		1	
Male	0.85	0.65-1.13	0.81	0.59-1.13
*Age*	1.00	0.99.-1.01	1.00	0.99-1.01
*Suicide prevention team*				
No	1		1	
Yes	1.16	0.90-1.50	0.90	0.67-1.22
*Previous attempt*				
No	1		1	
One	1.31	0.94-1.83	1.16	0.81-1.67
Several	2.75*	1.94-3.91	2.20*	1.50-3.24
*Aftercare referrals*				
Others	1		1	
Out-patient	1.55*	1.20-2.00	1.22	0.91-1.64
*Alcohol misuse*				
No	1		1	
Yes	2.02*	1.55-2.64	1.59*	1.14-2.22
*Marital status*				
Others	1		1	
Married/cohabitant	0.79	0.60-1.03	0.80	0.59-1.09
*Employment*				
Employed	1		1	
Unemployed/disabled	1.29	0.94-1.78	1.11	0.78-1.58
Student/pupil	0.46*	0.29-0.73	0.47*	0.28-0.79
Others	0.73	0.50-1.08	0.76	0.67-1.22

* p < 0.05

We also performed analyses including patients admitted to inpatient psychiatric care, (n = 1616 subjects). Assistance from the community team was given to a minority of the inpatient group. The results showed that receiving assistance from the suicide prevention team was not significantly associated with a repeated suicide attempt within 6 months (adjusted OR = 1.14; p > 0.05), 12 months (adjusted OR = 0.93; p > 0.05), or 5 years (adjusted RR = 0.94; p > 0.05) of follow-up.

Between 1984 and 2003, a total of 247 deaths among 1,422 individuals were recorded. Of these, 55 (22%) were suicide. Among the 1,422 individuals, it was possible to follow the risk of committing suicide within one year for 1,357 subjects, and within nine years for 895 subjects. No significant differences in suicide risk were observed between the two groups; the rate ratio (RR) was 0.33 (95% CI = 0.09-1.25) and 0.73 (0.36-1.48) at one and nine years of follow up, respectively. After adjustments for concurrent variables (Table 5), there was no significant difference in suicide risk between patients assisted by the suicide prevention team and patients who were not (adjusted RR = 0.85; 95% CI = 0.46-1.57). Age was significantly associated with suicide mortality: each year increased the risk for suicide by 1.03 times (95% CI = 1.01-1.04).

**Table 5:** Cox regression analysis of suicide mortality rates

Cox regression	Crude analysis		Adjusted analysis	
Variable	*RR*	95% CI	*RR*	95% CI
*Sex*				
Female	1		1	
Male	1.48	0.80-2.74	1.50	0.81-2.79
*Age*	1.03*	1.01-1.05	1.03*	1.01-1.05
*Suicide prevention team*				
No	1		1	
Yes	0.76	0.41-1.39	0.85	0.46-1.57
*Previous attempt*				
No	1			
One	1.56	0.72-3.39		
Several	0.93	0.47-3.95		
*Aftercare referrals*				
Others	1		1	
Out-patient	0.72	0.38-1.35	0.88	0.64-1.21
*Alcohol misuse*				
No	1			
Yes	1.07	0.51-2.23		
*Marital status*				
Others	1			
Married/cohabitant	1.28	0.67-2.48		
*Employment*				
Employed	1			
Unemployed/disabled	1.48	0.57-3.81		
Student/pupil	0.57	0.16-2.05		
Others	2.82*	1.19-6.66		

* p < 0.05

## Discussion

The objective of the present study was to investigate differences in the risk of repeated suicide attempt and/or suicide between individuals receiving additional assistance from a suicide prevention team or treatment as usual. There was no significant difference in repeat suicide attempt risk at 6 months, 12 months, or 5 years between individuals receiving suicide prevention team assistance or not. The results were similar on crude and adjusted analyses. There was also no significant difference in risk of successful suicide depending upon treatment.

Previous suicide attempts, marital status, and employment status were significantly associated with a repeated suicide attempt within both 6 and 12 months (p < 0.05). Individuals with a history of several suicide attempts prior to their index attempt had a more than double odds ratio of a repeated suicide attempt at both 6 months and 12 months. Being married/cohabitant and being a student/pupil were associated with reduced adjusted odds ratio of a repeated suicide attempt for the same time intervals. At 5-year follow-up, alcohol misuse and gender were significantly associated with repeated attempt, whereas marital status was non-significant. Male gender and being a student/pupil reduced the risk by 19% and 53%, respectively; several previous suicide attempts and alcohol misuse increased the risk by 2.20 and 1.59 times, respectively. Thus, in the present study, a history of previous suicide attempts was the strongest predictor for both short- and long-term repeated suicide attempts. Previous studies reported similar results [3,13,22]. Regarding suicide, only age was significantly associated with suicide mortality: the risk of death by suicide increased 3% for each year. Older age is a well-known risk factor reported in the literature for subsequent suicide in individuals with a history of suicide attempts [23,24].

The most accurate method to determine the efficacy of treatment modalities is via a randomized controlled trial. Due to ethical and practical reasons, the present study had a naturalistic design. The Bærum Model is based on individual evaluation of need, and assistance by the community suicide prevention team may not be relevant for all eligible patients. Thus, randomization to community team treatment or not would break with the clinical guidelines for the model and associated health care personnel. Consequently, patients were not randomized to either intervention or control in our study, nor did we have data on a comparable control group prior to the start of the intervention in 1983. The lack of control group in our study prevents us from providing a firm conclusion concerning the efficacy of this intervention model; we can simply state that there were no detectable differences in risk for repeated suicide attempts and successful suicide between individuals who did or did not receive assistance from the suicide prevention team. Regression analyses can only adjust for confounders, while selection bias can only be handled by randomization. Both self-selection by patients and selection by health care personnel may have biased our results. Patients were free to accept or decline assistance offered by the suicide prevention team. On the other hand, health care personnel could choose not to offer team assistance when circumstances deemed it inappropriate or unnecessary. On the other hand, in line with the philosophy of the Bærum model, one would expect that an efficient model of referral and care would result in patients - with or without the assistance of the community team - obtaining the same prognosis in terms of suicidal behavior repetition, either fatal or non-fatal.

Further, it was assumed that all interventions carried out at the hospital level were similar. As described above, a suicide prevention team including social workers and a psychiatrist is activated at the hospital immediately after emergency treatment is provided. All individuals who have attempted suicide and are admitted to a medical ward receive crisis intervention at the hospital. Appropriate measures are evaluated with the patient's cooperation before further referrals are made. This sequence of interventions may have *per se *a protective effect on future suicidal behavior; as a matter of fact, in Bærum the total incidence of repeated suicide attempts was 12% (95% CI = 10.7 to 14.1). This is significantly below the average repeated suicide attempt rate of approximately 15% reported in a systematic review by Owens et al. [3]. In the same study, the authors reported a suicide mortality rate between 0.5% and 2% one year after the suicide attempt and above 5% after nine years. The figures from Bærum were fairly similar: 1.1% (95% CI = 0.67-1.83) after one year and 4.6% (95% CI = 3.37-6.22) after nine years.

The positive outcomes described by Hvid and Wang [13] were from a clinical context that might be comparable to the Norwegian Bærum model. However, their study involved a quasi-experimental design, which was not possible to endorse in the present investigation. Perhaps in the future, in Norway, it would be feasible to compare the incidence of repeated suicide attempts in patients admitted to hospitals with and without a chain of care structure [25]. Variables such as treatment compliance [26] and quality of life should be controlled in order to verify their possible impact on chain of care interventions [25].

## Conclusions

This study did not detect significant differences in the risk of subsequent suicidal behavior between subjects whose aftercare involved a suicide prevention community-based service in addition to treatment as usual compared to those receiving treatment as usual only. While these results at first appear disappointing, it may actually indicate that the chain of care model is at least able to render indistinguishable - in terms of outcomes - subjects who require more intervention compared to those that do not. Of course, only a randomized, controlled trial could provide solid evidence of efficacy of the Bærum model. Within 12 months, an incidence of 12% for repeated suicide attempts was observed in the municipality of Bærum (psychiatric inpatients included), a figure slightly lower than the 15% reported in the literature. Whether this relatively lower incidence is related or not to the suicide prevention system in operation at hospital and community level remains unclear.

## Competing interests

The authors declare that they have no competing interests.

## Authors' contributions

HAJ made substantial contributions to conception and design of the manuscript, drafted the manuscript, analyzed and interpreted the data, and critically revised the manuscript for important intellectual content. GD was a central figure in organizing the Bærum Model. She has been in charge of data collection since 1984, and has substantially contributed to the draft of this paper. DDL made substantial contributions to conception and design of the manuscript, interpreted the data, and critically revised the manuscript for important intellectual content. BC critically revised the manuscript for important intellectual content. PHZ aided in data analysis and interpretation, and critically revised the manuscript for important intellectual content. All of the authors have read and approved the final manuscript.

## Pre-publication history

The pre-publication history for this paper can be accessed here:

http://www.biomedcentral.com/1471-2458/11/81/prepub
